# Attitudes Toward Injectable GLP‐1 Drugs for Obesity Treatment: A Qualitative Interview Study With Healthcare Professionals

**DOI:** 10.1002/osp4.70130

**Published:** 2026-03-12

**Authors:** Raluca C. Capaian, Phillippa Lally, Saskia C. Sanderson

**Affiliations:** ^1^ School of Psychology University of Surrey Guildford UK; ^2^ Department of Biostatistics and Health Informatics, Psychology and Neuroscience, King's College London Institute of Psychiatry London UK; ^3^ Department of Behavioural Science and Health University College London London UK; ^4^ NIHR Mental Health Translational Research Collaboration (MH‐TRC) Mission Oxford UK

**Keywords:** GLP‐1, interviews, obesity, qualitative, semaglutide, Wegovy

## Abstract

**Background:**

Injectable GLP‐1 drugs such as semaglutide (Wegovy) and tirzepatide (Mounjaro, a dual GLP‐1 and GIP receptor agonist) can help individuals living with obesity to lose weight. UK guidelines currently recommend semaglutide and other injectable GLP‐1 drugs are prescribed in specialist (Tier 3) multidisciplinary weight management services, and other settings. The study aimed to explore the views of healthcare professionals, working within a specialist weight management service that was not yet prescribing injectable GLP‐1 drugs, about these drugs being prescribed in their service in the future.

**Methods:**

In‐depth interviews were conducted with 11 healthcare professionals; thematic analysis was used to identify themes.

**Results:**

Thirteen main themes were identified within four theme clusters. *Knowledge*: (1) knowledge sources; (2) levels of current understanding. *Perceived benefits*: the drugs (3) might increase patients' confidence in their ability to lose weight and (4) provide a valuable middle ground treatment option between interpersonal behavioral support and bariatric surgery. *Concerns*: (5) patients might gain weight when they stop taking the drugs; the drugs might (6) mask behavioral patterns, (7) decrease patients' engagement with interpersonal behavior change support, and (8) have mental health risks. *Service provision considerations*: providing interpersonal behavior change support (9) before, (10) alongside, and (11) after stopping drug prescriptions were provided, (12) the drug prescription pathway is experimental, and (13) the role of healthcare professionals in specialist weight management services is evolving.

**Conclusions:**

The findings highlight several areas for further research and practice, including how specialist weight management service teams might integrate interpersonal behavioral support and injectable GLP‐1 drug prescriptions to best help patients in the future.

## 1 Introduction

Obesity is a worldwide epidemic, affecting one in eight people [1], which requires urgent attention from health authorities. In England, the National Health Service (NHS) weight management services pathway is organized in a 4‐tier framework [2, 3, 4, 5]: Tier one includes population‐level universal preventative care; Tier 2 services include time‐limited lifestyle (e.g., diet, exercise) interventions for individual patients mainly in primary care, but can also include commercial weight management services [6]; Tier 3 services are specialist multidisciplinary non‐surgical weight management clinics; and Tier 4 services provide bariatric surgery, including pre‐operative assessment and post‐operative care [5].

At the time the study was conducted, NICE recommended that injectable GLP‐1 drugs, such as semaglutide, should be offered to patients with obesity with a BMI of at least 35 kg/m^2^ plus at least one weight‐related comorbidity (or BMI 30–34.9 kg/m^2^ and meeting criteria for referral to Tier 3 or Tier 4 weight management services) [7]. Semaglutide can also be bought privately in the UK by patients outside the NHS [8, 9], and in January 2025, the NICE guidelines were updated so that tirzepatide, a dual GLP‐1 and glucose‐dependent insulinotropic polypeptide (GIP), could be prescribed by GPs within the NHS [10].

It is important to understand attitudes toward semaglutide among healthcare professionals responsible for supporting patients with obesity, including those supporting patients to make dietary and exercise behavioral changes [11]. A scoping review which included the attitudes of clinicians toward GLP‐1s showed that most studies focused on GLP‐1s as a treatment option for type 2 diabetes, and only two studies focused specifically on their weight‐loss utilization [12], one of which is an ethnographic study by Andreassen et al. [13]. This study examined how doctors and nurses (*N* = 11) in general practice in Denmark made decisions regarding semaglutide prescriptions [13]. The study suggested that healthcare professionals were generally trustful and had favorable views of semaglutide, in part because it has been used for diabetes management under the brand name Ozempic for several years, and they felt familiar with it. The healthcare professionals highlighted the importance of personal responsibility for weight loss and their reluctance to offer semaglutide to patients who wanted to lose weight mainly for aesthetic purposes. The healthcare professionals also mentioned the potential side effects and costs associated with semaglutide, but this did not lead to reluctance to prescribe it to patients [13]. In Denmark, the full cost of semaglutide is paid by patients, with healthcare professionals in primary care acting as gatekeepers. This differs from the UK, where NICE recommends that certain injectable GLP‐1 RA drugs, such as semaglutide, should be available to NHS patients via Tier 3 and other weight management services at no direct cost to patients [7], whereas tirzepatide can be prescribed within NHS primary care [10].

To date, no studies have investigated how healthcare professionals working in UK specialist weight management services view semaglutide to support the weight management of people with obesity [12]. The aim of the present study was therefore to explore attitudes (perceived benefits and concerns) and knowledge and information needs of healthcare professionals in a UK Tier 3 weight management service. The main research questions were as follows:

- What knowledge do healthcare professionals currently hold about injectable GLP‐1 drugs such as semaglutide, and do they feel sufficiently informed about their use in obesity management?
- What are healthcare professionals' views regarding the potential benefits of using injectable GLP‐1 drugs such as semaglutide in weight management?
- What concerns do healthcare professionals have regarding the use of injectable GLP‐1 drugs such as semaglutide in weight management?

## 2 Methods

### 2.1 Context

Healthcare professionals were recruited from a Tier 3 Weight Management service in England, which offers interpersonal nutritional advice and behavioral support to patients with a BMI of over 40 kg/m^2^ or a BMI between 35 and 39.9 kg/m^2^ if they also present with a weight‐related comorbidity such as Type 2 diabetes or hypertension. The Tier 3 weight management service was not yet offering semaglutide or any other injectable GLP‐1 drugs to patients when the interviews were conducted between 20^th^ June and 19th July 2024; however, applications were in place for approval to do so. Patients attending the service were offered 6 months of non‐surgical and non‐pharmacological support through interpersonal nutritional advice and behavioral support (e.g., dietary, exercise); patients who successfully lost at least 5% of their starting body weight within this timeframe, and were eligible for bariatric surgery in their geographical region, were then referred to a Tier 4 service. The Tier 3 service provided the interpersonal support for most patients remotely, via a combination of telephone and video appointments, with a minority of patients receiving in‐person appointments if they had special circumstances preventing them from attending video appointments.

### 2.2 Inclusion Criteria

Inclusion criteria were that the healthcare professionals were employed as a behavioral change advisor or nutrition advisor within this Tier 3 Weight Management service, none of whom had prescribing privileges.

### 2.3 Recruitment

The manager of the Tier 3 service agreed for staff to be invited to participate. Healthcare professionals were approached by one member of the research team who also worked as a behavioral change adviser within this service. Healthcare professionals were directly invited to take part in the study by email. The email included a link to an online survey, which displayed an information sheet, a written consent form (which participants signed electronically), a demographic questionnaire, and a list of potential interview dates for them to select from.

### 2.4 Design and Procedure

The interviews followed a semi‐structured schedule developed through discussion between the three authors, and adapted based on a pilot interview. The interview began with introductory questions about the participant and the Tier 3 service. This was followed by questions about the participants' knowledge about semaglutide, their views on its potential benefits for the patients they work with, and any concerns they might have about it. Participants were asked their views on whether the introduction of the drug could have psychological consequences, and whether patients receiving it should be advised differently to those not receiving it. The interview schedule also included questions on potential long‐term outcomes, how it might be integrated within the Tier 3 service, and their information needs. In line with good research practice (e.g., [14]), new questions were included to investigate specific areas of interest that emerged during the interviews. Whilst the interview schedule focused on attitudes toward semaglutide, interviewees' responses tended to relate to GLP‐1 drugs more generally. Interviews were audio‐recorded and transcribed verbatim. The data were analyzed according to Braun and Clark's [15] six‐stage framework, using a reflexive thematic approach. First, transcripts were read multiple times to allow data familiarization (stage 1). They were then systematically coded using software package NVivo 14, and preliminary themes were developed (Stages 2 and 3). Initial codes and themes were discussed and revised among all researchers, and a thematic map was created (Stage 4). The themes were defined and named (stage 5) and the report was then written (stage 6) [15].

The dataset was interpreted using a constructivist approach, which recognizes the significance of repetition, but places a higher importance on meaning and meaningfulness, which is socially formed and transmitted through a dynamic interaction between subjective and inter‐subjective development [16]. The analysis was conducted using a predominantly inductive approach [17]. However, an element of deductive approach was used to group themes into clusters to address the research questions [18].

### 2.5 Ethics

The study received favorable ethical approval from the University of Surrey's Ethics Committee [FHMS 23‐24 150 EGA].

## 3 Results

### 3.1 Participants and Recruitment

The recruitment email was sent to 17 healthcare professionals; 13 expressed interest in participating and 11 took part in an interview. Reasons for interviews not being conducted were related to scheduling restrictions. The sample size was decided using the theory of informationpower [19]. No new substantial themes were developed following consideration of the final interviews.

Six nutrition advisors and five behavioral change advisors participated: eight identified their ethnicity as White, 3 as Asian; nine identified as female, 2 as male. See Table 1 for further details. Interviews were conducted via Microsoft teams. Interviews lasted 34–62 min (mean duration 46 min).

**TABLE 1:** Participant characteristics.

Demographic characteristics (*n* = 11)		Number of participants
Age	Under 25 years	1
	Between 25 and 35 years	3
	Between 35 and 45 years	4
	Between 45 and 55 years	2
	Over 55 years	1
Gender	Female	9
	Male	2
Ethnic background	White	8
	Asian or British Asian	3
Role	Behavioral change advisor	5
	Nutrition advisor	6
Length of service	< 3 months	0
	3–6 months	2
	6 months to 1 year	3
	1 year to 3 years	3
	Over 3 years	3

### 3.2 Themes

#### 3.2.1 Knowledge and Information Needs About Injectable GLP‐1 Drugs

##### 3.2.1.1 Knowledge Sources

Participants had primarily gained knowledge about injectable GLP‐1 drugs through their observations of some of their patients who had bought the medication privately (Quote 1, Table 2). Participants' experiences with these patients shaped many of their views about semaglutide being available on the NHS within Tier 3 weight management services. Participants felt they had had little formal training about injectable GLP‐1 drugs, which had prompted some to seek information independently.

**TABLE 2:** Theme clusters, themes and example quotes.

Cluster of themes	Themes	Example quotes
Knowledge about injectable GLP‐1 drugs	Knowledge sources	*Quote 1*: “Is more kind of personal interest because we're coming across these people more often. Not not, you know, in a way, not… under our service, but within our service. So we're not, we're not prescribing this, but we are having to react to them on it.” (Participant 6)
	Levels of current understanding	*Quote 2*: “I don't know very much about it at this point. […] It would mean me going off and doing my own research and quite honestly, I haven't had the time at this point.” (Participant 2)
Perceived benefits of injectable GLP‐1 drugs	Injectable GLP‐1 drugs might increase patients' confidence in ability to lose weight	*Quote 3*: “I've seen that change already and it just from having the injectables and, you know, I think what it does is it gives the patient that little bit of a confidence boost ehm and sometimes that boost is what they need to then continue putting the hard work in because once they've lost a little bit of weight and, you know, they they want those good results to continue.[…] So, I think, you know, that there could be a real benefit in having injectables because it could be, you know, that that little glimmer of hope that people need.” (Participant 4)
	Injectable GLP‐1 drugs provide valuable middle ground between “lifestyle” behavior change support and bariatric surgery	*Quote 4*: “I think definitely, I think a weight loss injection pathway is needed because there are a lot of people who need help with weight loss but don't necessarily want to go as far as having bariatric surgery. So, I think this middle ground is a really positive pathway for patients.” (Participant 3)
Concerns about injectable GLP‐1 drugs	Patients might gain weight when they stop taking injectable GLP‐1 drugs	*Quote 5*: “I think it will be very interesting over time to see what happens when people come off it. I think my biggest concern is when people come off it uhmm. The potential to regain and whether that regain is more intense as with a lot of diets, we know that people regain at faster rate and more after they come off diets. And I worry that with coming off the medication this is going to happen again. So, is it a long‐term solution, a long‐term support? Or is it just a short‐term fix which is going to then create a bigger problem afterward?” (Participant 3)
	Injectable GLP‐1 drugs might mask behavioral patterns	*Quote 6*: “Again, I have had patients who… And this is patients primarily on Mounjaro, so I don't know if it's directly applicable, I know they are branches of the same drug. But I've noticed that patients are Monjaro have been able to eat… do less emotional eating, they've noticed just a reduction in that. But it's hard to know if that's because they were already doing this program and doing behavior change with with, with myself, and that they've been able to switch on to some of their emotional triggers a little bit more easily.” (Paticipant 9)
	Injectable GLP‐1 drugs might decrease patients' engagement with “lifestyle” advice	*Quote 7*: “I have had this before with patients who are losing the weight and then they kind of throw up their, their hands and say, yeah, it's all going well.” (Participant 1)
	Injectable GLP‐1 drugs might have mental health risks	*Quote 8*: “You know, someone that has maybe self‐harm tendencies, I I would be a bit concerned about giving it to this group of patients because, you know, once the injectable stops, you know, you know that could have a severe reaction, you know, a psychotic, you know, a mental health incident. So, I would err on the side of caution.” (Participant 4)
Service provision considerations for injectable GLP‐1 drugs	Providing “lifestyle” and behavior change support before prescribing injectable GLP‐1 drug	*Quote 9*: “I think rather than roll it out from the start of the program, I think a patient should be on the program for at least 3 months first, to show uhm changed behavior to begin with and then if they are on that right pathway of changed behavior and healthy eating habits, then they could access it. […] it could be good to limit limit it to try and stop people using it without the real emphasis on change of behavior and future.” (Participant 3)
	Providing “lifestyle” and behavior change support alongside prescribing injectable GLP‐1 drug	*Quote 10*: “But if it's used alongside other potentially more holistic measures that then get them put in place, you put in habit changes and use it to create that change, then yeah, it could be a really good solution.” (Participant 7)
	Providing “lifestyle” and behavior change support after stopping injectable GLP‐1 drug prescription	*Quote 11*: “There's a lot of concern about coming off a medication and that they're worried previous behaviors will return. […] I think some sort of intervention around the end when they're starting to titrate off may well be very useful for them so that they've got someone they can explore that with rather than just feeling they've got a GP that's just reducing their dosage.” (Participant 9)
	Injectable GLP‐1 drug prescription pathway seen as experimental	*Quote 12*: “I think in all honesty, that's something that we'd find out more when that time approaches, and we're actually doing the Wegovy injections.[…] I think it's something that is going to crop up when we're sort of in the process of doing it.[…] I think it's a learning process.” (Participant 2)
	The evolution of the role of health care professionals in Tier 3 weight management services	*Quote 13*: “They're losing the weight, they're really happy, there's nothing much that I can do here. What else? What…? What else is there that I can provide?” (Participant 5)

##### 3.2.1.2 Levels of Current Understanding

Most participants did not feel confident in their current understanding of injectable GLP‐1 drugs and thought they would benefit from more training, with only a minority considering themselves “fairly informed on it”. Lack of time was cited as a barrier to independent information seeking (Quote 2, Table 2).

All participants were aware regarding injectable GLP‐1 drugs, which can facilitate weight loss, although their understanding of the mechanisms of action varied. Most were aware that they induce weight loss through appetite suppression, and a few had deeper knowledge; for example, one participant mentioned that semaglutide works by mimicking the GLP‐1 hormone and was aware of some of the physiological effects of GLP‐1.

Most participants had some awareness of the eligibility criteria for injectable GLP‐1 drugs. They were aware that patients must have a BMI above a specified threshold and at least one weight‐related comorbidity. There was variation regarding how much detail participants provided about which comorbidities would make a patient eligible, though many mentioned diabetes.

Participants talked about various potential gastro‐intestinal side effects of injectable GLP‐1 drugs, such as nausea, constipation, and diarrhea.

#### 3.2.2 Perceived Benefits of Injectable GLP‐1 Drugs

##### 3.2.2.1 Injectable GLP‐1 Drugs Might Increase Patients' Confidence in Ability to Lose Weight

Most participants had positive attitudes toward injectable GLP‐1 drugs, describing them as a weight loss tool. Some felt injectable GLP‐1 drugs could increase patients' perceived behavioral control, giving them an initial confidence boost that would “kickstart” the weight loss process and encourage them to engage more with interpersonal behavioral support (Quote 3, Table 2). However, they also emphasized injectable GLP‐1 drugs are not a silver bullet and that patients should still put effort into the weight loss process.

##### 3.2.2.2 Injectable GLP‐1 Drugs Provide Valuable Middle Ground Between “Lifestyle” Behavior Change Support and Bariatric Surgery

A key theme was that many participants felt that “lifestyle” intervention alone was often not enough for this patient group. For many of them, patients' individuality was a decisive factor in their evaluation of injectable GLP‐1 drugs as obesity management tools, when compared to the non‐pharmacologically assisted “lifestyle” and behavior change intervention they currently provide, without one necessarily being “superior over the other”. Injectable GLP‐1 drugs were seen as a useful addition to standard care for patients with more complex needs, for example, hypothyroidism, polycystic ovarian syndrome, fibromyalgia, or reduced mobility and less ability to engage in physical activity. On the other hand, participants felt that losing weight by making “lifestyle” changes alone would be generally preferable if at all possible as it allows people to feel more “in control” of the process.

Injectable GLP‐1 drugs were also seen as providing a viable and preferable alternative to bariatric surgery for people who may need weight loss support beyond the interpersonal behavioral support intervention the participants currently provide, offering a “middle ground” for people who “don't necessarily want to go as far as having bariatric surgery” (Quote 4, Table 2). Participants saw prescribing injectable GLP‐1 drugs as a less invasive and more cost effective solution than bariatric surgery, and the fact that at the time of the interviews the NICE recommendation was that it would only be prescribed for 2 years was seen as an advantage by some.

#### 3.2.3 Concerns About Injectable GLP‐1 Drugs

##### 3.2.3.1 Patients Might Gain Weight When They Stop Taking Injectable GLP‐1 Drugs

A significant concern was what happens when patients stop taking injectable GLP‐1 drugs, with most worrying that patients would regain the weight lost while on the drugs, and possibly even more, as is the case following many weight loss attempts. That, in turn, could mean that people are “back to square one”, especially when the weight loss was not accompanied by behavioral changes, and because they might not be any closer to having developed a genuinely “healthier relationship with diets and food” (Quote 5, Table 2).

##### 3.2.3.2 Injectable GLP‐1 Drugs Might Mask Behavioral Patterns

Participants were also concerned that injectable GLP‐1 drugs could get patients in a “very positive bubble”, disguising some of the behavioral patterns they would normally work with their patients on, such as unhelpful habits, emotional eating, stress eating, and difficulties around prioritizing their health, making it harder to discern whether the “lifestyle” changes their patients are making are due to the advice they receive during interpersonal behavioral support, or the effects of the injectable GLP‐1 drug they take (Quote 6, Table 2).

##### 3.2.3.3 Injectable GLP‐1 Drugs Might Decrease Patients' Engagement With “Lifestyle” Advice

Participants worried that the weight loss produced by taking an injectable GLP‐1 drug could create a “false sense of security” in patients, causing them to “throw up their hands” (Quote 7, Table 2) and disregard the significance of following the behavior change guidance provided by their nutritionist and/or behavioral change advisor. They highlighted the potential for patients to think of injectable GLP‐1 drugs as a silver bullet, therefore distracting them from “the amount of work that they would have to put in to actually lose weight” by becoming “reliant on an external source to control their diet”.

##### 3.2.3.4 Injectable GLP‐1 Drugs Might Have Mental Health Risks

Some participants were concerned injectable GLP‐1 drugs could lead to mental health issues for some patients, for example, they worried that some patients might develop disordered eating patterns because of the appetite suppressive effects of the injectable drug. Some were also concerned injectable GLP‐1 drugs could exacerbate existing mental health problems or be a trigger for patients with a history of eating disorders, psychological trauma or self‐harm (Quote 8, Table 2).

#### 3.2.4 Service Provision Considerations for Injectable GLP‐1 Drugs

##### 3.2.4.1 Providing “Lifestyle” and Behavior Change Support Before Prescribing Injectable GLP‐1 Drug

Many participants suggested that before receiving an injectable GLP‐1 drug prescription, patients should spend time engaging with the “lifestyle” and behavior change advice provided by the multidisciplinary team. They believed this might give them a chance to assess each patient's individual needs and level of engagement, before the injectable GLP‐1 drug potentially “masks” behavioral patterns patients have struggled with thus far, as well as to have a conversation to manage expectations. They theorized that taking this approach could mitigate the risk of reduced engagement (Quote 9, Table 2). However, one participant pointed out that trying to assess a patient's willingness to make behavioral changes before they receive the injectable GLP‐1 drug prescription would leave things “more open for speculation”.

##### 3.2.4.2 Providing “Lifestyle” and Behavior Change Support Alongside Prescribing Injectable GLP‐1 Drug

Almost all participants felt that putting “more holistic measures” in place alongside the injectable GLP‐1 drug prescription “would be the most effective way to do it” (Quote 10, Table 2), which is consistent with NICE recommendations. There was some variation regarding the preferred level and frequency of this support, with some participants thinking they should be able to work with patients for the duration of them receiving the injectable drug prescription, while others thought “checking in” with patients regularly after the initial few months of support would be sufficient.

##### 3.2.4.3 Providing “Lifestyle” and Behavior Change Support After Stopping Injectable GLP‐1 Drug Prescription

Closely related to the risk of weight regain, some participants expressed their preference for patients to receive some support whilst titrating off the injectable GLP‐1 drug prescription and for a few months afterward, to help patients navigate their potential worries or challenges during the transition period, and potentially reduce the risk of weight regain (Quote 11, Table 2). However, a few of them mentioned this support would not necessarily have to come from within their service.

##### 3.2.4.4 Injectable GLP‐1 Drug Prescription Pathway Seen as Experimental

The introduction of an injectable GLP‐1 drug‐assisted weight loss pathway was seen by some participants as experimental. Some felt that working with patients prescribed injectable drugs will be “a learning process” before it will become evident how the service should be adapted to appropriately respond to patients' needs (Quote 12, Table 2).

##### 3.2.4.5 Evolution of the Role of Healthcare Professionals in Tier 3 Weight Management Services

Some participants were concerned about the potential increase in their level of responsibility, as patients might expect them to offer support relating to both the physical and psychological side effects of the medication, issues which they felt went beyond their role and training.

Many participants reflected on how to adapt their support and advice to patients taking a GLP‐1 drug, emphasizing the need to “encourage them to think about the long term” and guide patients toward considering how they might cope once they stop taking the GLP‐1 drug. They generally felt the advice would be similar to the advice they currently give patients not receiving a GLP‐1 prescription, “just framed a little bit differently”.

On the other hand, the potential risk of patients becoming reliant on injectable GLP‐1 drugs for weight loss made some participants question the usefulness of their professional input regarding “lifestyle” and behavior change support (Quote 13, Table 2). This concern was especially mentioned regarding cases where an injectable GLP‐1 drug might mask behavioral patterns that participants would normally work on with their patients.

As the NHS has already approved the prescription of injectable GLP‐1 drugs within Tier 3 weight management services, participants mentioned that numerous patients come onto the program expecting to receive it, which meant they had to manage the patients' disappointment at not receiving an NHS GLP‐1 drug prescription and to redirect patients' focus to “lifestyle” and behavior change support interventions alone, which at many times was challenging.

Please refer to Table S1 for additional quotes supporting the Themes presented above.

## 4 Discussion

Healthcare professionals participating in this study expressed a range of views regarding the use of injectable GLP‐1 drugs such as semaglutide in weight management for people living with obesity. Participants felt injectable GLP‐1 drugs could help patients if used to facilitate “lifestyle” and behavioral change; they also saw the drugs as a valuable middle ground solution for patients for whom interpersonal “lifestyle” and behavior change support alone might not be enough but who are ineligible or choose not to opt for bariatric surgery.

Participants' concerns about injectable GLP‐1 drugs for obesity management related to their perceptions that the drugs could potentially become a “quick fix”; they expressed concern that the appetite suppressive properties could mask some behavioral patterns, leading to patients engaging less with interpersonal “lifestyle” and behavior change support, and ultimately weight regain.

Both perceived benefits and concerns have stirred discussion around service provision. Participants made suggestions about the order in which patients should receive interpersonal “lifestyle”, behavior change support, and injectable GLP‐1 drug prescriptions to maximize the likelihood of weight loss and minimize the likelihood of weight re‐gain. They also discussed how introducing injectable GLP‐1 drugs into their service might alter their current roles and responsibilities.

The findings in this study are consistent with and extend existing evidence on this topic. Firstly, healthcare professionals in other studies also highlighted the importance of using GLP‐1 RAs as a supplement to interpersonal behavioral support to change healthy eating habits and increase the amount of exercise [13]. In a qualitative study investigating the perspectives of GPs who are working within the NHS in England on the use of GLP‐1 RAs for obesity management, Keating et al. [20] reported that GPs saw the drugs as a positive “tool in the toolbox” but one that could potentially distract from addressing the psychological, behavioral, social, and commercial determinants of obesity. In a survey conducted in the United States (US) on healthcare professionals' perceptions of anti‐obesity medication, the majority (73%) agreed that these medications are useful to “kickstart” patients' weight loss, and almost half (43%) agreed that ultimately anti‐obesity medication should only be a short‐term solution [21].

Secondly, participants in both the present study and Andreassen et al.’s [13] felt they had to, or will have to, learn as they go about how to best support patients receiving injectable GLP‐1 drug prescriptions. Similar to the Danish healthcare professionals, participants in this study felt they had not yet received sufficient information from health authorities on this matter (which in the Danish study had led the healthcare professionals to develop their own in‐clinic service provision guidelines). A cross‐sectional survey conducted in the US with primary‐care physicians, endocrinologists, and advanced practice providers (physician assistants/associates and nurse practitioners) who were current prescribers of anti‐obesity medication to patients with obesity found that only 23% of primary‐care physicians, and 21% of advanced practice providers, considered themselves “extremely familiar” with GLP1‐RAs, GIPs, and glucagon receptor agonists [22].

It is worth noting that the participants interviewed in this study did not have prescribing privileges and that their patients were not prescribed weight loss medications as part of the service they offered at the time. Therefore, they had to rely on a combination of “what if” scenarios and experience of working with patients buying injectable GLP‐1 drugs privately. In contrast, healthcare professionals in other prior studies, who did have experience with prescribing GLP‐1 drugs, were less likely to see the introduction of these drugs in clinical practice as experimental. For example, participants in Andreassen et al.’s study noted a “fundamental trust in the science of medicine” and familiarity with this class of drugs as they have used it for the treatment of diabetes for a number of years. This fits with evidence that the more familiar healthcare professionals were with this class of drugs, the more they were willing to recommend them to their patients without diabetes to support weight loss [22].

In the present study, participants’ perceptions of the effects of injectable GLP‐1 drugs (including semaglutide) on their patients partially fit with findings from randomized clinical trials (RCTs). For example, participants in this study noted that their patients reported reduced emotional (over)eating whilst taking injectable GLP‐1 drugs; this is consistent with the findings of a study in which semaglutide was associated with decreased emotional eating and external eating (eating in response to external food cues), both of which are associated with increased BMI [23]. The psychological intervention component of the Tier 3 weight management service our participants provide addresses emotional eating and external eating. The reduction in these obesity‐associated eating patterns’ results may reflect changes in neural pathways involved in food intake and reward [24], which could arguably result in psychological advice becoming non‐essential. Participants in this study questioned how their role would change when patients receive an injectable GLP‐1 drug prescription within their service, worrying that patients taking a GLP‐1 drug might engage less with their interpersonal “lifestyle” and behavior change support. Future research should investigate the extent to which patients prescribed injectable GLP‐1 drugs also comply with, value, and benefit from interpersonal “lifestyle” and behavioral support.

At the time of the interviews, semaglutide was approved by NICE [7] for a maximum of 2 years; participants therefore questioned what will happen next, and how injectable GLP‐1 drugs (including semaglutide) can be integrated into current standard of care to avoid benefits being temporary. Semaglutide was approved for use in obesity management following the five STEP trials [25]. These trials investigated the effects of semaglutide on individuals living with obesity when prescribed alongside a “lifestyle” (i.e., diet and physical activity) intervention. The STEP 1 trial 1‐year follow‐up showed that after semaglutide was discontinued, participants regained two‐thirds of the weight they had lost [26]. Moreover, the STEP 4 study found that after a 20‐week run‐in period of semaglutide, which resulted in a mean weight loss of 10.6%, participants who were switched to placebo and stopped the lifestyle intervention had a mean body weight increase of 6.9% from Week 20 to Week 48 [27]. Therefore, due to concerns about weight regain after stopping injectable GLP‐1 drugs, future research might usefully investigate whether combining an interpersonal “festyle” and behavior change intervention alongside injectable GLP‐1 drug prescriptions leads to a significantly greater reduction in weight compared to the drugs being prescribed without or with less interpersonal support. Additionally, future research should focus on determining the optimal structure and content of interpersonal “lifestyle” and behavior change interventions to maximize short‐ and long‐term benefits for patients.

Some participants expressed concern about the potential adverse effects of injectable GLP‐1 drugs on mental health. However, evidence suggesting this is a substantial risk is lacking: in contrast, one retrospective cohort study found that administering semaglutide to patients with binge eating disorder (BED) actually led to greater decreases in Binge Eating Scale scores compared to two other commonly prescribed anti‐obesity drugs used to treat BED [28].

Finally, some participants expressed concern about injectable GLP‐1 drugs being prescribed to patients with self‐harm tendencies, suggesting that disappointment about having to eventually stop taking the drug could lead to a severe mental health incident. However, following investigations, the U.S. Food and Drug Administration (FDA) and European Medicines Agency concluded that current evidence does not support claims of a causal relationship between injectable GLP‐1 drugs and “suicidal and self‐injurious thoughts and actions” [29, 30]. In fact, a meta‐analysis [31] showed a significant improvement in depression scores in patients treated with a GLP‐1 compared to alternative anti‐diabetic treatments. Research is ongoing assessing both the benefits of, and potential risk factors for experiencing adverse responses to, injectable GLP‐1 drugs; as evidence is gathered, further stakeholder interviews will also valuably provide insights on information needs and support policymakers in creating more targeted guidelines.

Strengths of this qualitative study include that this is the first research to investigate healthcare professionals' attitudes toward the use of injectable GLP‐1 drugs such as semaglutide in the weight management of people living with obesity in the UK. The main limitation of this study is that it was conducted within a single Tier 3 weight management service, and therefore, the findings are not necessarily generalizable. Participants were working in a healthcare service clinic setting not yet prescribing injectable GLP‐1 drugs. Future research in the UK should investigate whether stakeholder attitudes differ when eligible patients receive NHS‐funded injectable GLP‐1 drug prescriptions within weight management services.

Lastly, the researcher who conducted the interviews was known to the participants and working within the same service participants were recruited from; this familiarity could have influenced participants to express their views more openly, but might also have constrained the interaction.

## 5 Conclusion

Healthcare professionals in this qualitative interview study expressed their views on injectable GLP‐1 drugs for weight loss, perceiving benefits and concerns in complex ways. The findings highlight areas for further investigation, and particularly highlight the need for further policy‐relevant research into how best to combine interpersonal behavioral support (or “lifestyle” and behavior change) interventions along with weight‐loss injectable drugs for maximum effectiveness. Weight‐loss injectables have important financial implications for the NHS and the patients it serves, both those living with obesity and other citizens [32, 33]. It is estimated that around 1.6 million people in the UK are currently taking a GLP‐1 drug for weight management [34], and this number could potentially increase to as many as 3.4 million in the upcoming years following the approval of tirzepatide (brand name Mounjaro) in December 2024 [10, 35]. Understanding the views of healthcare professionals in services such as NHS Tier 3 programs in the UK can play a vital role in informing obesity management policy and practice going forward.

## Author Contributions

R.C., P.L., and S.C.S. together designed the research study including materials. R.C. collected and analyzed the data. All authors were involved in writing the paper and had final approval of the submitted and published version.

## Funding

The authors have nothing to report.

## Conflicts of Interest

Dr. Saskia Sanderson is on the Scientific Advisory Board for the Danish Precision Health Initiative (also known as DELPHI), led by Professor Ruth Loos, University of Copenhagen, Novo Nordisk Foundation Center for Basic Metabolic Research, Copenhagen.

## Supporting information


**Table S1:** Theme clusters, themes and example quotes.

## Data Availability

Research data are not shared.
