# Supplementary material for: Attitudes Toward Injectable GLP‐1 Drugs for Obesity Treatment: A Qualitative Interview Study With Healthcare Professionals
Source: Obes Sci Pract. 2026 Mar 12;12(2):e70130. doi: 10.1002/osp4.70130 (PMC13494737; doi:10.1002/osp4.70130)
Supplement: Supplementary file 1 [file OSP4-12-e70130-s001.docx]

Supplementary Table 1. Theme clusters, themes and example quotes

|  | **Themes** | **Example quotes** |
| --- | --- | --- |
| **Knowledge about injectable GLP-1 drugs** | Knowledge sources | "Nothing's been sort of given to me in terms of information from my my workplace" (Participant 2) |
|  | Levels of current understanding | "So in there were like exclusion criteria and inclusion criteria, but it was for like people who are having BMI about 35. And…I think for diabetes and hypertension" (Participant 10)  "There's some side effects like constipation, nausea, diarrhoea..." (Participant 11)  "I I only know very little definitely, but I think it helps you lose the weight. The target is that it suppresses your appetite…? Yeah, it's an injection that helps you lose the weight because it helps you suppress the appetite. Yeah, that's all I know." (Participant 5) |
|  |  | "I think I'm…[pause] reasonably informed on it, really 'cause it's just… I find it interesting so I I yeah… I'd like to think I'm fairly informed on it, yeah." (Participant 8) |
| **Perceived benefits of injectable GLP-1 drugs** | Injectable GLP-1 drugs might increase patients’ confidence in ability to lose weight | "You need to do it yourself. It's a helping hand, like a training wheel, but it it doesn't solve everything. [...]My ideas from where I stand, I think that it it may be helpful to kickstart. You know, as a word that patients quite like to use to kick start the weight loss or help kind of trigger things.[...] It needs maintenance still with lifestyle changes, but it just maybe sorts out their metabolism to the point where they can lose weight as long as they put in all the work, let's say. (Participant 1) |
|  | Injectable GLP-1 drugs provide valuable middle ground between ‘lifestyle’ behaviour change support and bariatric surgery | "Obviously, it would be great if no one ever needs a medical intervention to lose weight, but the patients we see are here for a reason. They're here because they struggle with their weight, they're here because they've struggled with weight loss in the past. I don't think we’re… going to be able to get every single patient to lose weight through non-medical change, or the amount of weight that they do need to to lose." (Participant 7) |
|  |  | "But the benefit is, as opposed to surgery, it's not something that…It's not as invasive to take this medication, so that's a a huge plus for people" (Participant 1)  "But weight weight loss surgery is expensive whereas I I suspect that the people could just go on an injection to lose weight, it’s gonna be a lot more cost effective to do it that way." (Participant 2)  "I agree with the idea that it should only be used for a limited period of time. I think it's one of the big advantages to it is, that it's a temporary measure." (Participant 7) |
| **Concerns about injectable GLP-1 drugs** | Patients might gain weight when they stop taking injectable GLP-1 drugs | "I think it will be very interesting over time to see what happens when people come off it. I think my biggest concern is when people come off it uhmm. The potential to regain and whether that regain is more intense as with a lot of diets, we know that people regain at faster rate and more after they come off diets. And I worry that with coming off the medication this is going to happen again. So, is it a long-term solution, a long-term support? Or is it just a short-term fix which is going to then create a bigger problem afterwards?” (Participant 3) |
|  | Injectable GLP-1 drugs might mask behavioural patterns | "When they're having the injectables and they're seeing the weight loss, that they tend to [...] be in this very positive bubble at the time." (Participant 4) |
|  | Injectable GLP-1 drugs might decrease patients’ engagement with ‘lifestyle’ advice | "It's like an easy fix almost and, you know, seeing those weight loss results just from taking an injectable it it it kind of deviates from, you know, the amount of work that they would have to put in to actually lose weight, you know, if they weren't having the injectables and and the amount of work that they might, they will probably have to put in after finishing the injectables. So, I I think sometimes it's a false sense of security, I guess, that they get from the injectables." (Participant 4)  "It does concern concern me because there's it a, it's a reliance on an external source to control their diet essentially. So, in a way it's like well, it doesn't matter whether you're going for a like a milkshake diet or a cabbage soup diet or a medication. It's an external source that you're given the control to rather than being able to manage it yourself. So, I do think so I think it's concerning" (Participant 11) |
|  | Injectable GLP-1 drugs might have mental health risks | "Perhaps just building a bit of a, you know, an unhealthy relationship with food, if the appetite's reduced by a large amount. [...] And if there's any eating disorders, maybe as well from the past, you know, with the nausea or throwing up, that might be a bit triggering as well for those patients." (Participant 11)  "With a small subset of my patients…uhm…Loss and and sometimes rapid loss can cause a lot of fear. And I would say especially in patients who are looking at trauma backgrounds. So if you're looking at patients that previous been…previously been in abuse backgrounds, who were probably starved by partner due to abuse or or raped by a family member or as a child. They will definitely start to find some quite serious psychological effects of losing weight rapidly." (Participant 9) |
| **Service provision considerations for injectable GLP-1 drugs** | Providing ‘lifestyle’ and behaviour change support before prescribing injectable GLP-1 drug | "From the behaviour change point of view, is I would have the initial appointment before they'd even started taking medication, so you can actually work out psychologically what those issues are with a patient." (Participant 9)  "I think you could, but I think it leaves it more open for speculation. [...] I think if you're starting to leave it up to the behaviour change advisors or nutrition advisors to decide who does get it and who doesn't, you end up with issues with favouritism, you end up, end up with issues of some people giving it while others wouldn’t. And I don't think that level of kind of subjectivity is a good thing" (Participant 7) |
|  | Providing ‘lifestyle’ and behaviour change support alongside prescribing injectable GLP-1 drug | "And they benefit a lot for from it, for their weight loss journey. But only…I'd say that only if they have the complementary lifestyle…ehm…Support as well from a healthcare professional that's… I feel like that would be the most effective way to do it.[...]I've also noticed that's where the weight loss really comes in there. When they're both kind of complimenting each other in the treatment and the lifestyle advice, I think that shows really good results." (Participant 11) |
|  | Providing ‘lifestyle’ and behaviour change support after stopping injectable GLP-1 drug prescription | “After the two years? Yeah. Oh, absolutely. I think they should be checked in on and I would suggest that be primary care or their GP basically…I think.” (Participant 8) |
|  | Injectable GLP-1 drug prescription pathway seen as experimental | "I feel that the roll out of this medication is a bit of an experiment. It's a bit of a trial like, it it has been trialled, but on a larger scale, like this it's a larger trial with the community." (Participant 1) |
|  | The evolution of the role of health care professionals in Tier 3 weight management services | "I don't want to get too involved in it because I'm not a medical practitioner to an extent that I would prescribe medication. So, if I knew too much, I don't have...the full education behind prescribing medications, so I don't want to know it too in depth.And give away too much information without having a qualification behind me that supports that." (Participant 3)  "And then are they prepared for those things when they happen? And again, you know that's… you know, if we're there to support them with that, that's not really behaviour change, that's psychological therapy work, [pause] Which is slightly different. I know we do both. But you know what I mean? It's not necessary our remit." (Participant 6)  "I would encourage them to think about the long term and to, you know, take the advice on board for what they need to be doing after they come off the injectables. And also, you know, putting some of it into place now to, you know, to support that weight loss journey. And I would, I guess, I would sell it to them as a long-term strategy for weight loss. But the advice would still be the same. It's just framed a little bit differently." (Participant 4)  "But you're not really dealing with any overeating habits…uhm…Potentially any comfort eating that they might have been doing is now gone because actually they feel too sick to eat it anyway. So it's it's almost like it's… Can you work with them while they're on the injection? Because that's masking, or, you know… Helping with with with the issues." (Participant 6)  "And you have to sort of… it's very difficult to say “Well, I'm sorry, but we don't do this. But we do this. Do you want this?” You know? But they're so set on, you know, the injection's gonna be the answer. And the doctor said we can give it to them. And then we're saying we can't. Is this like…uhm…It's not not easy to have to back that about all the time." (Participant 6) |
|  |  |  |
|  |  |  |
|  |  |  |
